# Influence of professional experience and work schedule on sleep quality

**DOI:** 10.47626/1679-4435-2025-1428

**Published:** 2025-07-23

**Authors:** Matheus de Souza Dias, Michael Jackson Oliveira de Andrade

**Affiliations:** 1 Laboratório de Neurociências, Cronobiologia e Psicologia do Sono, Universidade do Estado de Minas Gerais, Divinópolis, MG, Brazil

**Keywords:** circadian rhythm, sleep quality, shifts work, rotating., ritmo circadiano, qualidade do sono, trabalho em turnos.

## Abstract

**Introduction:**

Extended work hours have been associated with negative impacts on workers’ physical and mental health, including stress, fatigue, and sleep disorders. Night shift workers or those with irregular schedules are particularly affected due to circadian rhythm disruptions, which impair sleep quality and occupational health.

**Objectives:**

To investigate the sleep-wake patterns of daytime and nighttime shift workers by analyzing sleep efficiency, latency, total sleep duration, and frequency of nighttime awakenings.

**Methods:**

A longitudinal exploratory study was conducted with 15 workers (mean age = 27.93; standard deviation = ± 8.50) in Divinópolis, Minas Gerais. The instruments used were the Pittsburgh Sleep Quality Index, the Insomnia Severity Index, and actigraphy. Data collection was carried out using actigraphy for 7 consecutive days, covering the entire workweek, including weekends.

**Results:**

Workers with over 10 years of experience showed higher insomnia severity, lower sleep efficiency, and a greater number of awakenings (p < 0.05). Morning shift workers had better sleep patterns, with longer duration and higher efficiency compared to afternoon/night shift workers.

**Conclusions:**

Sleep quality among workers is influenced by professional experience, time in the role, and work schedule. Adjustments in working conditions that consider circadian rhythms and chronotype are essential for improving sleep health and productivity. Sleep management strategies should be implemented to promote well-being and performance.

## Abstract (pt)

**Introdução:**

Jornadas de trabalho prolongadas têm sido associadas a impactos negativos na saúde física e mental dos trabalhadores, incluindo estresse, fadiga e distúrbios do sono. Trabalhadores noturnos ou em turnos irregulares são particularmente afetados devido a alterações nos ritmos circadianos, prejudicando a qualidade do sono e a saúde ocupacional.

**Objetivos:**

Investigar os padrões de sono e vigília de trabalhadores em turnos diurnos e noturnos, analisando a eficiência do sono, latência, duração total e frequência de despertares noturnos.

**Métodos:**

Realizou-se um estudo longitudinal exploratório com 15 trabalhadores (média = 27,93; desvio padrão = ± 8,50) em Divinópolis, Minas Gerais. Foram utilizados como instrumentos: Índice de qualidade do sono de Pittsburgh, Escala de gravidade de insônia e o instrumento actígrafo. A coleta de dados foi feita por meio de actigrafia durante 7 dias consecutivos, abrangendo toda a semana de trabalho, incluindo finais de semana.

**Resultados:**

Trabalhadores com mais de 10 anos de experiência apresentaram maior gravidade de insônia, menor eficiência do sono e maior número de despertares (p < 0,05). Os trabalhadores do turno da manhã tiveram melhores padrões de sono, com maior duração e eficiência do sono em comparação aos turnos tarde/noite.

**Conclusões:**

A qualidade do sono dos trabalhadores é impactada pela experiência profissional, tempo no cargo e horário de início da jornada. Ajustes nas condições de trabalho, considerando os ritmos circadianos e o cronótipo, são essenciais para melhorar a saúde do sono e a produtividade. Estratégias de gestão do sono devem ser implementadas para promover bem-estar e desempenho.

## INTRODUCTION

The World Health Organization (WHO) and the International Labour Organization (ILO) emphasize that prolonged working hours significantly contribute to the global burden of health conditions, compromising workers’ physical and mental health as well as their overall well-being.^1^ Evidence shows that excessive workloads are directly associated with increased stress and fatigue in the workplace - conditions that raise the risk of problems such as anxiety and depression.^2,3^ The impact of extended working hours on workers’ health is not limited to psychological and emotional aspects; it also involves complex biological mechanisms that affect sleep regulation and overall bodily function. These effects are further exacerbated by the growing prevalence of long working hours and irregular shifts, which expose a substantial portion of the workforce to schedules misaligned with the human biological rhythm.^3^

Sleep-wake regulation is based on the interaction between two main processes: Process S (homeostatic), which increases the propensity for sleep during wakefulness due to the accumulation of adenosine in the brain, and Process C (circadian), which is controlled by the central biological clock located in the suprachiasmatic nucleus and adjusts the sleep-wake cycle to a 24-hour pattern.^4^ Overall, this model emphasizes that the interaction between homeostatic and circadian mechanisms not only regulates the timing and intensity of sleep but also ensures effective recovery and hormonal regulation during sleep.^5,6^ Disruptions in circadian rhythms and mismatches between circadian phase and diurnal preference are critical factors in maintaining sleep quality.^7,8^

Variability in work schedules - especially irregular shifts - causes significant disturbances to biological rhythms, leading to adverse consequences such as excessive sleepiness,^5^ sleep disorders,^6^ increased symptoms of anxiety,^9,10^ depression, substance use, cognitive deficits, and reduced quality of life.^11,12^ These risks are directly linked to circadian rhythm disruption and sleep deprivation.^13,14^ Shift workers are particularly vulnerable to various sleep disorders, the most common being shift work sleep disorder,^15,16^ insufficient sleep syndrome,^17,18^ and obstructive sleep apnea.^17^ Moreover, sleep deprivation is recognized as a significant risk factor for several occupational diseases, affecting complex biological processes involving oxidative stress and inflammatory responses, impairing immune function, and contributing to cardiovascular and neurodegenerative diseases.^19,20^

Night work leads to a major disruption in circadian rhythms, negatively impacting sleep patterns and quality, resulting in difficulty initiating sleep, reduced sleep duration, and increased sleepiness during shifts.^3,21,22^ The main objective of this study was to investigate sleep efficiency (SE) as well as behavioral patterns related to sleep among workers, considering different types and schedules of work shifts. Specifically, we aimed to examine how variations in working hours, including day and night shifts, affect sleep quality, sleep onset latency (SL), total sleep duration, and the frequency of nighttime awakenings.

## METHODS

### STUDY LOCATION

The study was conducted in the city of Divinópolis, Minas Gerais (20°S, 44°W), located in the southeastern region of Brazil, where sunlight duration shows significant seasonal variation. Data collection took place between June and December, during which the average relative humidity was 64%, and the mean temperature was 21°C. The average duration of sunlight during this period was 8.52 hours per day, with an average of 112 minutes of rainfall per day throughout the study months.

### STUDY DESIGN

This was an exploratory study with a longitudinal design, in which sleep-wake patterns and light exposure were continuously monitored using a wrist-worn actigraphy device on the dominant hand for 7 consecutive days (168 hours). The monitoring period included 5 weekdays and 2 weekend days. Participant data were analyzed separately for weekdays and non-weekdays, considering the variables of total professional experience, time in the current job, and current work start time.

### PARTICIPANTS

Initially, 23 adults were recruited for the study. However, only 15 participants completed all phases of the research. The final sample consisted of 15 volunteers (10 women and 5 men), with a mean age of 27.93 years [median (Mdn) = 24.5, standard deviation (SD) = ± 8.50, range: 19-51 years]. Participants included both daytime and nighttime shift workers, with an average daily work schedule of 9.33 hours (SD = ± 1.72, range = 6-12 hours) and a mean weekly rest time of 1.80 days (SD = ± 0.67, range = 1-3 days). All volunteers were free of clinical or neurological diseases and did not report anxiety-related behaviors or significant levels of weekly stress.

### INSTRUMENTS

Sociodemographic and clinical questionnaire: An online questionnaire composed of openand closed-ended questions designed to collect relevant information on sociodemographic, clinical, and work-related aspects of the volunteers. It included the following categories: social information (name, sex, gender, educational level), clinical history (COVID-19, medical treatment, neuropsychiatric disorder, sleep disorder, prescription medication, visual system), work conditions (work shift, working hours, work schedule), family background, and physical activity.

Pittsburgh Sleep Quality Index (PSQI): a self-administered tool consisting of 19 questions regarding sleep quality and disturbances over the past month. PSQI was developed by Buysse et al. and validated in Brazil in an adult population by Bertolazi et al.^23^ The questionnaire assesses seven components of sleep: subjective quality, sleep latency, sleep duration, SE, sleep disturbances, use of sleeping medication, and daytime dysfunction. Each component is scored from 0 to 3, with a maximum total score of 21 points.

Insomnia Severity Index: a brief self-assessment questionnaire consisting of seven items that evaluate the nature, severity, and impact of insomnia on a 5-point Likert scale (0-4), with total scores ranging from 0 to 28 points. The cutoff points for classifying insomnia severity are as follows: absence of significant insomnia (0-7), subthreshold insomnia (8-14), moderate clinical insomnia (15-21), and severe clinical insomnia (22-28).^24^

Actimeter: the ActTrust 2 - an advanced wristwatch-style actimeter designed to measure physical activity, light, and temperature from both the user and the environment - was used. ActTrust 2 includes spectral light and temperature sensors and allows for event marking, such as medication intake. The device also includes ActStudio software for analyzing the collected data.^25^

### PROCEDURES

This study was approved by the Research Ethics Committee of the Universidade Federal da Paraíba (approval number 2.777.645). Participation was entirely voluntary and formalized through the signing of an informed consent form, in accordance with Resolutions No. 466/12 and 510/16 of the Brazilian National Health Council (Ministry of Health). Workers were recruited through social media and directed to an online form hosted on the Google Forms platform. In this form, participants agreed to the terms of the informed consent and completed a sociodemographic and clinical questionnaire.

Participants were invited to the study site at a previously scheduled date and time. During this visit, they received detailed instructions on how to use the actimeter, which was to be worn for 7 consecutive days, covering the entire workweek, including weekends. The device was worn continuously, 24 hours a day, throughout the data collection period. After returning the actimeters, participants completed a second online form that included the PSQI and the Insomnia Severity Index.

### OUTCOME VARIABLES

The outcome variables in this study included behavioral sleep measures: main rest phase (FTS), total sleep time (TTS), SE, SL, wake after sleep onset (WASO), number of awakenings (nWASO), secondary sleep time in naps (ST), and total sleep duration over 24 hours (TSD24).

### STATISTICAL ANALYSIS

The Mann-Whitney test was used for comparisons between independent measures, considering the different groups on weekdays and non-weekdays. The Wilcoxon test was applied for paired measures. All statistical analyses were performed using IBM SPSS Statistics for Windows, version 22.0 (IBM Corp., Armonk, N.Y., USA), with a p-value less than 0.05 considered statistically significant.

## RESULTS

The participants were predominantly White (57.1%), had completed higher education or more (57.1%), reported a family income between two and four minimum wages (42.9%), were single (71.4%), and held only one job (78.6%). On average, they had 9.8 years of work experience (SD = ± 7.6) and had been in their current job for an average of 33.80 months (SD = ± 32.20). Regarding the overall score for subjective perception of sleep quality, although participants with greater work experience and longer time in their current job reported poorer sleep quality (Mdn = 8.8; 95% CI: 6-9.57) compared to those with less work experience and shorter job tenure (Mdn = 7.63; 95% CI: 5-9), this difference was not statistically significant, either for work experience (p = 0.330) or for time in the current job (p = 0.304).

**Figure 1:**
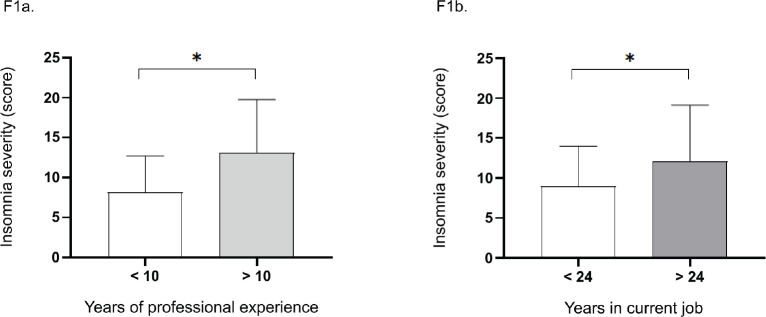
Insomnia severity index according to years of professional experience and time in the current job in a sample of Brazilian workers.

However, the data indicate that individuals with more than 10 years of professional experience had a higher insomnia severity index (Mdn = 13.74; 95% CI: 11.28-15) compared to those with less than 10 years of experience (Mdn = 9.82; 95% CI: 3-13), with a statistically significant difference (U = 2.0, z = 2.843, p = 0.003, r = 0.54). Similar results were observed for time in the current job: workers with more than 24 months of employment showed higher insomnia scores (Mdn = 13.48; 95% CI: 7-15) compared to those with less than 24 months (Mdn = 10.65; 95% CI: 3-13), also with a statistically significant difference (U = 7.5, z = 2.175, p = 0.026, r = 0.41). Data analysis is shown in Figure 1.

### VARIATIONS IN SLEEP QUALITY BY GENDER AND OCCUPATIONAL FACTORS

The study included 10 women (Mdn = 23; 95% CI: 19-25) and five men (Mdn = 27.5; 95% CI: 19-51). Although women had a higher median and a broader confidence interval, the groups did not differ significantly for the analyzed variable, indicating homogeneity (p = 0.055). No significant difference was found regarding years of professional experience (p = 0.513); however, a significant difference was observed for time in the current job (p = 0.028), with women (Mdn = 33; 95% CI: 8-108) having longer tenure compared to men (Mdn = 7; 95% CI: 1-60). Regarding sleep quality, data showed that women had longer TTS (Mdn = 6h46min) compared to men (Mdn = 6h23min), with a statistically significant difference (U = 3.0; z = 2.40; p = 0.014; r = 0.30). No significant differences were observed for FTS (p = 0.076), SE (p = 0.945), SL (p = 0.945), WASO (p = 0.539), nWASO (p = 0.539), ST (p = 0.999), or TSD24 (p = 0.203). Additionally, no significant differences were found for overall sleep quality (p = 0.635) or presence of insomnia-related problems (p = 0.454).

### PROFESSIONAL EXPERIENCE: ANALYSIS ON WEEKDAYS AND WEEKENDS

A significant difference was found in years of professional experience (U = 36.0, z = 3.137, p = 0.002, r = 0.56), with longer work experience observed in individuals with more than 10 years of experience (Mdn = 13.50; 95% CI: 10-16) compared to those with less than 10 years (Mdn = 5; 95% CI: 4-7).

Regarding weekdays, the Mann-Whitney test revealed significant differences for SE (U = 3.0, z = 2.71, p = 0.005, r = 0.72), nWASO (U = 7.00, z = 2.19, p = 0.029, r = 0.59), and ST (U = 0.01, z = 3.24, p < 0.001, r = 0.87). Participants with more than 10 years of professional experience showed lower SE (Mdn = 88.95; 95% CI: 88.12-89.26), fewer WASO (Mdn = 7.46; 95% CI: 5.00-8.40), and greater time spent napping (Mdn = 23.00; 95% CI: 14.13-44.00) during weekdays. On weekends, significant differences were also observed for nWASO (U = 8.00, z = 2.07, p = 0.043, r = 0.55) and SL (U = 8.50, z = 2.05, p = 0.043, r = 0.55).

Individuals with more than 10 years of professional experience also showed more WASO (Mdn = 11.00; 95% CI: 6.33-16.50) and longer SL (Mdn = 1.75; 95% CI: 0.00-3.00) on weekends.

When behavioral sleep variables were analyzed on weekdays and weekends among workers with more than 10 years of professional experience, the Wilcoxon test showed significant differences for TTS (z = 2.20, p = 0.028), nWASO (z = 1.99, p = 0.046), ST (z = 2.20, p = 0.028), and TSD24 (z = 2.20, p = 0.028). These workers showed longer TTS (Mdn = 444.34; 95% CI: 391.67-550.33), more WASO (Mdn = 11.00; 95% CI: 6.33-16.50), longer nap duration (Mdn = 54.26; 95% CI: 41.83-64.15), and longer total sleep over a 24-hour period (Mdn = 500.62; 95% CI: 433.50-592.83) on weekends compared to weekdays. In contrast, for workers with less than 10 years of professional experience, the Wilcoxon test revealed significant differences for WASO (z = 2.10, p = 0.036), ST (z = 2.52, p = 0.012), and TSD24 (z = 2.38, p = 0.017). Participants had longer ST (Mdn = 54.84; 95% CI: 10.67-95.67) and greater TSD24 (Mdn = 481.50; 95% CI: 425.17-556.50) on weekends, whereas WASO was higher on weekdays (Mdn = 44.10; 95% CI: 30.25-64.00). Data are presented in Table 1.

**Table 1:** Sleep variables on WD and WE according to total professional experience in a sample of Brazilian workers

Sleep	Group 1 (≤ 10 years)				Group 2 (> 10 years)				U	p-value
	M ± SD (min)	Mdn (min)	Min (min)	Max (min)	M ± SD (min)	Mdn (min)	Min (min)	Max (min)		
WD										
FTS	443.75±31.4	451.48	377.75	485.80	445.60±34.6	446.50	409.25	495.00	20.00	1.000
TTS	414.45±33.5	407.27	383.80	482.20	402.87±22.0	399.00	384.00	438.60	15.00	0.282
SE	90.38±1.0	90.31	88.66	91.80	88.72±0.5	88.95	88.12	89.26	3.00	0.005^*^
SL	1.38±0.8	1.37	0.33	2.75	2.00±1.8	1.00	0.25	4.50	19.00	0.573
WASO	45.57±10.6	44.10	30.25	64.00	38.98±13.5	36.50	25.00	55.40	17.00	0.414
nWASO	9.21±1.7	9.79	6.00	11.20	7.02±1.3	7.46	5.00	8.40	7.00	0.029^*^
ST	4.95±3.9	4.86	0.00	12.11	27.39±12.5	23.00	14.13	44.00	0.01	0.000^*^
TSD24	405.33±25.7	408.72	356.86	444.80	440.88±34.7	450.50	403.90	488.00	16.00	0.345
WE										
FTS	449.56±38.4	456.00	383.00	501.50	499.29±55.5	481.84	447.00	583.67	14.00	0.228
TTS	416.32±32.6	423.75	363.50	449.00	453.23±56.4	444.34	391.67	550.33	15.00	0.282
SE	92.86±2.9	93.00	89.00	97.00	90.50±3.3	90.50	87.00	94.00	14.50	0.228
SL	0.57±0.7	0.25	0.00	2.00	1.75±1.0	1.75	0.00	3.00	8.50	0.043^*^
WASO	29.66±16.1	30.00	8.50	54.00	35.04±11.1	35.11	19.00	49.33	19.00	0.573
nWASO	7.30±2.3	8.00	3.50	10.50	11.25±3.5	11.00	6.33	16.50	8.00	0.043^*^
ST	50.05±28.3	54.84	10.67	95.67	53.09±9.6	54.26	41.83	64.15	24.00	1.000
TSD24	483,52±49,0	481,50	425,17	556,50	510,74±58,2	500,62	433,50	592,83	16,00	0,345

FTS = main rest phase; Max = maximum; M = mean; Mdn = median; Min = minimum; min = minute; nWASO = number of awakenings after sleep onset (WASO); ST = secondary sleep time in naps; SD = standard deviation; SE = sleep efficiency; SL = sleep onset latency; TSD24 = total sleep duration over 24 hours; TTS = total sleep time; WD = weekdays; WE = weekends.

### TIME IN CURRENT JOB: ANALYSIS ON WEEKDAYS AND WEEKENDS

A significant difference was found in time in the current job (U = 28.0, z = 3.137, p = 0.002, r = 0.56), with longer job tenure observed in individuals with more than 24 months of employment (Mdn = 60.00; 95% CI: 30.0-108.0) compared to those with less than 24 months (Mdn = 8.00; 95% CI: 1-24).

On weekdays, the Mann-Whitney test indicated a significant difference for ST (U = 2.00, z = 2.84, p = 0.003, r = 0.76). Participants with more than 24 months in their current job had longer nap durations (Mdn = 26.63; 95% CI: 11.13-44.13) compared to those with less than 24 months. No statistically significant differences were found between the two groups on weekends (p > 0.05).

When comparing weekdays and weekends among workers with more than 24 months of job tenure, a significant difference was found only for ST (z = 2.20, p = 0.028), with longer nap durations on weekends (Mdn = 54.00; 95% CI: 41.83-104.00) compared to weekdays. For workers with less than 24 months in their current job, significant differences were found for both ST and TSD24 (z = 2.37, p = 0.018). These participants had longer ST (Mdn = 55.67; 95% CI: 10.67-193.00) and longer TSD24 (Mdn = 475.50; 95% CI: 425.17-556.50) on weekends compared to weekdays (Table 2).

**Table 2:** Sleep variables on WD and WE according to time in current job in a sample of Brazilian workers

Sleep	Group 1 (≤ 24 months)				Group 2 (> 24 months)				U	p-value
	M ± SD (min)	Mdn (min)	Min (min)	Max (min)	M ± SD (min)	Mdn (min)	Min (min)	Max (min)		
WD										
FTS	454.02±49.1	448.80	377.75	537.40	440.76±33.2	431.75	33.16	409.25	21.00	0.710
TTS	414.23±36.3	405.37	383.80	482.20	391.54±12.5	392.63	12.49	373.15	18.00	0.456
SE	90.27±1.1	90.09	88.66	91.80	89.77±1.4	89.28	1.45	88.25	19.00	0.535
SL	1.53±0.3	1.40	0.60	2.75	2.42±1.9	2.25	1.97	0.25	24.00	1.000
WASO	46.32±11.2	47.20	30.25	64.00	39.82±11.8	40.25	11.85	25.00	16.00	0.318
nWASO	9.01±1.7	9.68	6.00	11.20	7.51±1.7	7.25	1.75	5.00	17.00	0.383
ST	8.56±7.9	5.71	0.00	18.60	28.00±11.1	26.63	11.13	14.13	2.00	0.003^*^
TSD24	408.30±10.7	409.60	387.80	422.31	425.60±22.6	420.76	22.65	403.90	16.00	0.318
WE										
FTS	445.86±39.5	455.00	383.00	501.50	485.33±159.8	494.00	171.00	683.00	14.00	0.180
TTS	413.79±34.4	420.50	363.50	449.00	433.88±139.7	448.67	152.00	576.00	15.00	0.225
SE	93.00±3.2	94.00	89.00	97.00	89.57±3.8	89.00	84.00	94.00	11.50	0.091
SL	1.21±1.8	0.50	0.00	5.00	1.50±1.2	1.50	0.00	3.00	18.50	0.432
WASO	28.43±16.9	24.00	8.50	54.00	49.47±30.2	43.33	19.00	107.00	14.00	0.180
nWASO	7.14±2.5	8.00	3.50	10.50	10.78±5.3	13.33	5.00	17.33	15.00	0.224
ST	67.41±62.7	55.67	10.67	193.00	62.70±23.2	54.00	41.83	104.00	22.00	0.749
TSD24	481.19±52.5	475.50	425.17	556.50	496.58±124.7	501.57	256.00	630.00	16.00	0.277

FTS = main rest phase; Max = maximum; M = mean; Mdn = median; Min = minimum; min = minute; nWASO = number of awakenings after sleep onset (WASO); ST = secondary sleep time in naps; SD = standard deviation; SE = sleep efficiency; SL = sleep onset latency; TSD24 = total sleep duration over 24 hours; TTS = total sleep time; WD = weekdays; WE = weekends.

### WORK START TIME: ANALYSIS ON WEEKDAYS AND WEEKENDS

Descriptive analysis showed that the group starting work in the morning began at 7:57 a.m. (SD = 0.71) and ended at 5:23 p.m. (SD = 1.38). The afternoon/evening group started at 2:18 p.m. (SD = 4.85) and finished at 11:33 p.m. (SD = 6.03). Data indicate significant differences on weekdays for SE (U = 1.00, z = 2.87, p = 0.002, r = 0.77), ST (U = 4.00, z = 2.47, p = 0.012, r = 0.66), and TSD24 (U = 1.00, z = 2.87, p = 0.002, r = 0.77) as well as a significant difference in SL on weekends (U = 7.00, z = 2.12, p = 0.042, r = 0.56). As presented in Table 3, morning-shift workers showed higher SE, longer nap duration, and longer total sleep duration compared to afternoon/evening-shift workers, both on weekdays and weekends. The only exception was NT, which was greater among afternoon/evening workers on weekends.

**Table 3:** Sleep variables on WD and WE according to work shift start time in a sample of Brazilian workers

Sleep	Group 1 (7 a.m.-9 a.m.)				Group 2 (10 a.m.-10 p.m.)				U	p-value
	M ± SD (min)	Mdn (min)	Min (min)	Max (min)	M ± SD (min)	Mdn (min)	Min (min)	Max (min)		
WD										
FTS	441.18±32.2	446.50	377.75	495.00	442.27±33.8	448.80	407.32	485.80	22.00	1.000
TTS	396.85±25.5	399.00	344.75	438.60	395.98±31.5	384.00	360.49	443.80	19.00	0.699
SE	90.20±1.3	90.09	88.25	92.33	85.97±3.15	86.92	80.86	88.66	1.00	0.002^*^
SL	2.00±1.4	1.80	0.33	4.50	0.90±0.5	0.80	0.25	1.50	10.00	0.112
WASO	42.45±9.6	41.00	28.75	55.40	44.28±14.6	44.00	25.00	64.00	21.00	0.898
nWASO	8.82±1.8	9.34	6.00	11.20	7.82±1.9	8.00	5.00	10.17	16.00	0.438
NT	15.34±7.5	18.20	2.50	25.28	3.37±2.9	4.00	0.00	7.19	4.00	0.012^*^
TSD24	433.36±27.3	430.68	404.10	487.91	394.77±15.2	403.11	371.20	407.83	1.00	0.002^*^
WE										
FTS	481.11±59.7	471.00	383.00	583.67	436.60±30.5	447.50	401.33	469.67	13.00	0.240
TTS	443.55±52.3	448.67	369.50	550.33	407.00±40.5	420.00	363.50	447.00	10.00	0.112
SE	92.52±3.5	94.00	87.00	97.00	89.92±1.3	89.00	89.00	92.00	11.50	0.147
SL	1.50±1.1	1.85	0.00	3.00	0.15±0.2	0.00	0.00	0.47	7.00	0.042^*^
WASO	33.05±19.4	30.33	8.50	69.00	36.27±13.6	36.00	19.00	54.00	19.00	0.699
nWASO	9.78±5.1	8.00	3.50	17.33	6.90±1.8	7.50	5.00	9.00	17.50	0.518
NT	56.13±22.4	54.00	20.83	95.67	90.91±63.0	67.50	28.50	193.00	12.00	0.190
TSD24	507.48±77.8	499.67	425.17	630.00	495.30±38.2	487.50	455.41	556.50	22.00	1.000

SD = standard deviation; WD = weekdays; TSD24 = total sleep duration over 24 hours; SE = sleep efficiency; WE = weekends; FTS = main rest phase; SL = sleep onset latency; M = mean; Max = maximum; Mdn = median; Min = minimum; min = minute; nWASO = number of awakenings after sleep onset (WASO); ST = secondary sleep time in naps; TTS = total sleep time.

Regarding behavioral sleep data for morning-shift workers on weekdays and weekends, significant differences were found for TTS (z = 2.19, p = 0.028), ST (z = 2.67, p = 0.008), and TSD24 (z = 2.07, p = 0.038). Participants spent more time in bed, took longer naps, and had greater total sleep duration over 24 hours on weekends compared to weekdays. Similarly, the Wilcoxon test showed significant differences for SE, SL, ST, and TSD24, all with z = 2.02 (p = 0.043) for the afternoon/evening-shift group. As shown in Table 3, SE (Mdn = 89.00; 95% CI: 89.00-92.00), ST (Mdn = 67.50; 95% CI: 28.50-193.00), and TSD24 (Mdn = 487.50; 95% CI: 455.41-556.50) were higher on weekends, while SL was longer on weekdays (Mdn = 0.80; range = 0.25-1.50).

## DISCUSSION

The results of this study show that factors such as professional experience and length of time in the current job stand out as significant determinants of sleep quality, including variables such as efficiency, latency, and total sleep duration, as well as insomnia severity. Moreover, the differences observed between sleep patterns on weekdays and weekends highlight adjustments related to occupational demands, with important implications for workers’ health. In addition, gender-related factors may significantly influence work dynamics and sleep patterns, as suggested - albeit preliminarily - by the findings of this study.

Although a trend toward poorer sleep quality was observed among individuals with greater professional experience and longer time in their current job, these differences did not reach statistical significance. However, workers with more than 10 years of experience showed significantly higher insomnia severity, as did those with more than 24 months in their current job. These findings indicate that longer exposure to work is associated with a negative impact on sleep quality.

Despite apparent contradictions among the analyzed categories, the scales used to measure sleep quality and insomnia severity have adequate validity and reliability for the studied population. Nevertheless, fluctuations in the results may be attributed to the self-reported nature of the scales, which can introduce subjective biases or recall difficulties - especially among workers exposed to high workloads. Moreover, the sleep quality scale used does not account for factors such as nighttime awakenings or sleep regularity, which may limit data interpretation. On the other hand, the use of objective measures such as actigraphy provided more robust data, contributing to a better understanding of these differences.

The results revealed significant differences between groups with up to 10 years and more than 10 years of professional experience across various dimensions of sleep. Individuals with up to 10 years of experience showed lower SE on weekdays and a higher nWASO, a pattern that may be related to lower adaptation to occupational stressors typical of the early stages of a career, as suggested by the studies of Lyons et al.^26^ and Burgard & Ailshire,^27^ which discuss how work intensity and stress negatively affect sleep quality. On the other hand, workers with more than 10 years of experience showed lower SE on both weekdays and weekends, appearing to be more affected by the cumulative effects of chronic stressors, including rotating shifts, night work, and long working hours, all of which may lead to circadian disruption and negatively impact SE.^3,7,21,22^

Additionally, individuals with more than 10 years of professional experience reported longer sleep duration and more napping on weekends, which may indicate an attempt to compensate for accumulated nighttime sleep deficits over the course of their professional trajectory. This compensation may function as a strategy to mitigate the effects of inadequate nighttime sleep during weekdays, aiming to restore physiological balance and reduce fatigue.

These results may be attributed to circadian changes related to the flexibility of work schedules during time off. Reduced schedule rigidity on weekends, combined with decreased occupational demands, can destabilize the biological rhythms established throughout the week, leading to longer SL and more frequent nighttime awakenings. As a result, variations in sleep schedules may affect the continuity and quality of rest, making it more difficult for the body to adapt to a regular and healthy rhythm - especially after exposure to irregular or demanding work shifts during the week.^8,9^

Job tenure also emerged as a relevant factor in our study. On weekends, both groups showed an increase in ST and TTS, suggesting that sleep deprivation accumulated throughout the week generates a “sleep debt” that needs to be “paid off” during rest days. This behavior can be interpreted as the body’s attempt to restore the homeostatic balance of sleep, as proposed by the sleep homeostasis regulation theory.^5^ In addition, increased sleep may also reflect an effort to adjust circadian rhythms and reestablish synchronization between physiological needs and sleep patterns.^6,7^

Findings related to the influence of work start time on sleep patterns support studies on circadian rhythms, chronotype, and individual differences.^28,29^ Workers whose shifts began around 7:57 a.m. (SD = 0.71) exhibited better sleep quality, longer total sleep duration, and more time spent napping compared to those starting around 5:23 p.m. (SD = 1.38). This can be explained by a greater alignment between their work hours and natural circadian rhythms.

Chronotype, or the biological preference for being more active during the day or at night, plays a significant role in adapting to work schedules. Individuals with a morning chronotype adapt more easily to early shifts, as these schedules align with their biological rhythms. Conversely, evening-type workers tend to struggle with morning shifts, which can lead to sleep disturbances and reduced sleep quality. Factors such as age and gender also influence these responses, with variations observed according to age group and the specific physiological needs of men and women.

## CONCLUSIONS

Based on the results of this study, we suggest that workers’ sleep quality is influenced by a range of occupational and individual factors, such as professional experience, length of time in the current job, and work start time. Workers with longer careers and more time in their current positions tend to show poorer sleep quality, longer sleep latency, and a greater need for daytime naps, suggesting that the accumulation of occupational stressors over time impairs circadian rhythms and physiological recovery. One limitation of this study is the relatively small sample size, composed of workers from a single region of Brazil. This geographical restriction may limit the generalizability of the findings to other occupational groups and gender-related issues in different socioeconomic and cultural contexts - especially in cities with varying population dynamics and labor conditions.

Work start time also has a significant impact, as workers who begin their shifts in the morning display better sleep quality indicators, likely due to better alignment with their natural circadian rhythms. These results highlight the need to adjust working conditions to promote employee well-being by considering individual physiological needs such as chronotype.

Furthermore, implementing strategies for sleep management - including efforts to reduce sleep debt and promote psychoeducation on sleep hygiene and circadian health - should be a priority for improving both health and productivity in the workplace. Adopting such approaches may help promote healthier, more restorative sleep, with positive effects on job performance and workers’ overall well-being.
